# Effectiveness of Regulatory Policies on Online/Digital/Internet-Mediated Alcohol Marketing: a Systematic Review

**DOI:** 10.1007/s44197-023-00088-2

**Published:** 2023-02-02

**Authors:** Sandra Radoš Krnel, Gorazd Levičnik, Wim van Dalen, Giulia Ferrarese, Sandra Tricas-Sauras

**Affiliations:** 1 Analysis and Development Centre, National Institute of Public Health, Trubarjeva Cesta 2, 1000 Ljubljana, Slovenia; 2 EUCAM - European Centre for Monitoring Alcohol Marketing, Utrecht, The Netherlands; 3 Ecole de Santé Publique, Centre de Recherche Approches Sociales de la Santé, Brussels, Belgium; 4 Eurocare: The Alcohol Policy Alliance, Brussels, Belgium

**Keywords:** Alcohol, Regulation, Digital, Marketing, Advertising, Effectiveness, Policy

## Abstract

**Background:**

The rapid growth of social networking sites and video sharing platforms has created an opportunity for the alcohol industry to employ advanced advertising and marketing approaches to target their audiences, increasingly blurring the lines between commercial marketing and user-generated content, which poses a challenge for effective regulation.

**Methods:**

We conducted a systematic search through three peer-reviewed journal databases (WoS, PubMed, Scopus). Studies were included if published in English, after 2004, and assessed statutory regulation or voluntary industry codes, enacted by an EU or nation’s governmental agency or private entity, and with the intent to restrict digital alcohol advertising. In addition, we conducted a manual search of gray literature.

**Results:**

A total of 4690 records were identified. After duplicate removal and full-text assessment, 14 articles were examined. Our findings indicate that children and adolescents may often be exposed to alcohol advertisements on social media and websites due to industry’s self-regulatory age-affirmation systems being largely ineffective at preventing under-aged access. Cases of self-regulatory violations by the alcohol industry, and increasingly innovative ‘gray-area’ advertising approaches have also been noted. Additionally, research illustrates a lack of developed statutory restrictions of digital alcohol advertising and instead continued reliance on voluntary industry self-regulation.

**Conclusions:**

There is a substantial need for further research to examine the effectiveness of digital alcohol advertising restrictions in social media, websites and image/video sharing platforms. Moreover, there is a necessity for countries to develop comprehensive statutory frameworks, which would effectively restrict and monitor rapidly advancing digital alcohol advertising practices on new digital media.

**Supplementary Information:**

The online version contains supplementary material available at 10.1007/s44197-023-00088-2.

## Introduction

Alcohol use has been identified as one of the most important risk factors for the global burden of mortality and disease, having been associated with over 200 adverse medical conditions and having caused over 3 million deaths worldwide in 2016 [1]. The latest data from the World Health Organization (WHO) places the WHO European Region as the geographical region with the world's highest level of total alcohol consumption, with the lowest prevalence of abstainers in the population, and as the region with over 10% of all deaths having been caused by alcohol-attributing factors in the year 2016, including one out of four deaths among young adults aged 20–24 years [2]. Because of the public health impact, numerous efforts have been made to identify risk factors that lead to excessive alcohol use. One such risk factor is exposure to alcohol marketing [3]. Evidence for the association between exposure to alcohol marketing and alcohol use among young people is growing. Recent longitudinal studies show that young people with higher levels of exposure to marketing are more likely to initiate alcohol use and consume alcohol in harmful patterns [4].

Since the past decade, we are continuously witnessing the gain of popularity of various online platforms or social networking/image sharing sites. In light of said growth, alcohol advertisers have shifted their focus to digital media, which unlike advertising through traditional one-way communication channels, provide the alcohol industry the ability to advertise content by a multitude of contemporary marketing approaches. Marketing through these new media channels can be targeted at specific audiences, virally spread between users, and accessed in almost any context (e.g., via smartphones), and can actively recruit users into the marketing process [5]. Users are therefore actively engaged with alcohol brands’ content through “liking” and commenting on ads or pictures, sharing, retweeting, following other users or by conversing with them on brand web pages, or by posting their own branded images [6–9]. Social networking sites serve as a public forum in which young people actively value, identify with and make use of alcohol marketing messages [10]; therefore, it is in an alcohol brand's interest to build its values and identity by establishing positive and desirable associations with their social media followers. This type of marketing communication increasingly blurs the lines not only between entertainment and commercial messages, but also between personal communication and commercial messages [11].

A study analyzing the amount of digital alcohol advertising which young people are exposed to in four European member states, found that of the participating 9380 school students aged 10–18, over 65% self-reported seeing alcohol advertisements on internet pages, over 33% reported receiving promotional e-mails mentioning alcohol brands, and over 18% reported downloading a mobile phone or computer screensaver containing an alcohol brand name or logo [12]. Two more recent studies from the USA also found that a substantial amount of under-aged young people was exposed to digital alcohol advertising content [9, 13]. A recent systematic review by Noel et al. confirmed that engagement with digital alcohol marketing, same as in case of exposure to alcohol marketing through traditional media channels, is positively associated with increased alcohol consumption and increased binge or hazardous drinking behaviors [3].

Regulation of the marketing of alcoholic beverages, including content and volume, is an important instrument for reducing alcohol consumption, and is recognized as a WHO “best buy” policy measure [5]. Introducing bans or partial restrictions is a cost-effective policy measure to reduce consumption and associated harm [14]. Despite the ample evidence to suggest that digital marketing may be more powerful and less controllable than traditional alcohol marketing, many governments have yet to consider placing an emphasis on implementing statutory digital alcohol marketing regulations. According to WHO’s Global Status Report on Alcohol and Health [1], less than half of EU member states self-reported having at least a partial alcohol marketing ban on internet websites and social networking sites in the year 2016. Effective monitoring and restricting digital alcohol advertising content on the internet and social media are complexed by a number of challenges. One prominent issue facing EU and national regulators is the fact that digital marketing activities transcend national borders [15], making it extremely difficult to assign an element of geography to such advertising activity due to its inherent global reach [16]. As highlighted by the newest 2022 WHO Technical Report [17], cross-border alcohol marketing continues to present a profound regulatory challenge for most EU member states, and a growing proportion of alcohol is produced and marketed by transnational alcohol companies who are also among the highest advertising and promotion spenders worldwide. Another regulatory issue also presents itself in lieu of accelerated growth of alcohol purchase and delivery websites, which facilitate closer links between alcohol producers and their customers, increase the availability of alcohol and have already been identified as often easily bypass-able by under-aged youth, who in multiple cases were able to order and have alcohol beverages delivered to their assigned addresses [18–20]. The utilization by alcohol brands of various new “stealth marketing” tactics (product placements, creation of new media profiles, channels, brand names, graphical designs and/or slogans with the intent for said digital elements to closely resemble the alcohol brand’s original corporate identity) [21], and the emergence of the so-called *social influencers*, who often have a strong influence particularly on young people, can significantly shape a customer’s purchasing decision [22, 23], thereby imposing additional pressure to regulators. As overwhelmingly recognized by contemporary alcohol marketing research literature [3, 24–30], there is an ever-growing necessity for countries to develop comprehensive regulations, governing alcohol marketing on new digital media such as online video sharing/hosting platforms, or social networking/image sharing sites.

In light of the above, we conducted a systematic review of the available scientific and authoritative gray literature to determine if existing statutory regulation as well as voluntary industry self-regulation in restricting online/digital/internet-mediated alcohol marketing can be considered as effective.

## Methods

The systematic review main search run was conducted throughout the months of May and June of 2021 through (1) Scopus, (2) Web of Science and (3) PubMed journal databases. The following Boolean search string of keywords was used to identify potentially eligible articles: (marketing OR promot* OR advert*) AND (digital OR online OR internet) AND (alcohol*) AND (regulat* OR monitor* OR polic* OR supervis* OR enforc* OR restrict*). After exporting the initial article list, an eligibility assessment of the gathered studies was performed independently in a standardized manner by at least three researchers. Those reviewed the titles and abstracts of each article to determine if it met the inclusion and exclusion criteria for the subsequent full-text assessment. We manually searched (for further content) on the web addresses of peer-reviewed journals, such as Alcohol, Alcohol and Alcoholism, Journal of Studies on Alcohol and Drugs, and Addiction. We also searched for relevant authoritative gray literature through various international, governmental, or independent research organizations' websites (e.g., World Health Organization, European Commission’s Public Health Portal, European Alcohol Policy Alliance, European Centre for Monitoring Alcohol Marketing).

To reduce bias of chosen articles, increase reliability and transparency, and improve the communication of the findings, a systematic review process was selected using the 2009 PRISMA (Preferred Reporting Items for Systematic Reviews and Meta-Analyses) statement reporting approach. We used the Covidence© online primary screening and data extraction tool as a platform to conduct the majority of our systematic review. A methodological protocol was submitted and registered on PROSPERO under the serial: CRD42021244848. The draft of the protocol was subject to prior review by two experts with relevant research experience in the field of digital alcohol advertising.

### Eligibility Criteria

We restricted the eligible article publishing dates from the year 2004–present as it marks the first year of placed online advertisements being implemented by a major social networking site and/or video hosting/sharing platform [31], as well as the beginning of gradual worldwide adoption of such digital technologies, which were deemed the predominant focus of our systematic review. Studies were consequently included if they analyzed the impacts and/or assessed the effectiveness of any forms (statutory regulation, co-regulation, self-regulation) of digital alcohol advertising restrictions or regulatory frameworks on any social networking/image sharing site, online video hosting/sharing platform or regular website as well as e-mails, downloadable content e.g., screensavers, and mobile phone apps. Studies were excluded from further assessment if their abstracts or titles were not published in English language. If any article was found with both the title and abstract in English, but with the rest of the text written in a foreign language, we contacted the respective authors for a translated version. Studies which did not assess digital alcohol advertising content, were excluded from the full-text assessment. Additionally, records were excluded if being a non-primary research data source, e.g., an editorial, review, opinion piece, or a book.

### Study Selection and Data Extraction

We followed the Noel et al. [3] definition of digital alcohol advertising as alcohol-branded content on websites, media pages and banner advertisements on social networking sites and video sharing platforms, e-mails, and downloadable content e.g., screensavers, and mobile phone apps. Effectiveness of digital alcohol advertising regulations was adapted from Burton et al. [14] and defined as the degree to which a country's enacted statutory regulations, or social networking service providers’ online video hosting platforms’, social networking/image sharing sites’ or regular websites’ self-regulatory alcohol advertising rules and/or codes of practice, or as any combination of the aforementioned forms as part of a co-regulatory framework, reduce the public health burden of alcohol. The types of research study designs chosen to be included in our review were all types of observational, experimental studies, retrieved from selected online research databases, conducted at a national, regional, or local level, including other secondary and/or gray literature (policy evaluation documents, reports, audits, etc.), gathered from authoritative sources, such as governmental, municipal, or specialized agencies' websites or independent research institutions.

### Quality Assessment and Risk of Bias

An adapted version of the Hawker et al. [32] Study Quality Assessment Tool was used for general study quality appraisal, the scoring domains having included Abstract and Title, Introduction and Aims, Method and Data, Sampling, Data Analysis, Ethics and Bias, Findings/Results, Transferability/Generalizability, Implications and Usefulness (Appendix 1). The process was conducted by two researchers with any disagreements resolved by a third one. We did not exclude any articles from the review, based solely on the score from the quality appraisal. In the cases where a study did not involve any human participants and instead solely analyzed various datasets, the sampling methods and ethics would be scored adaptively.

## Results

**Fig. 1:**
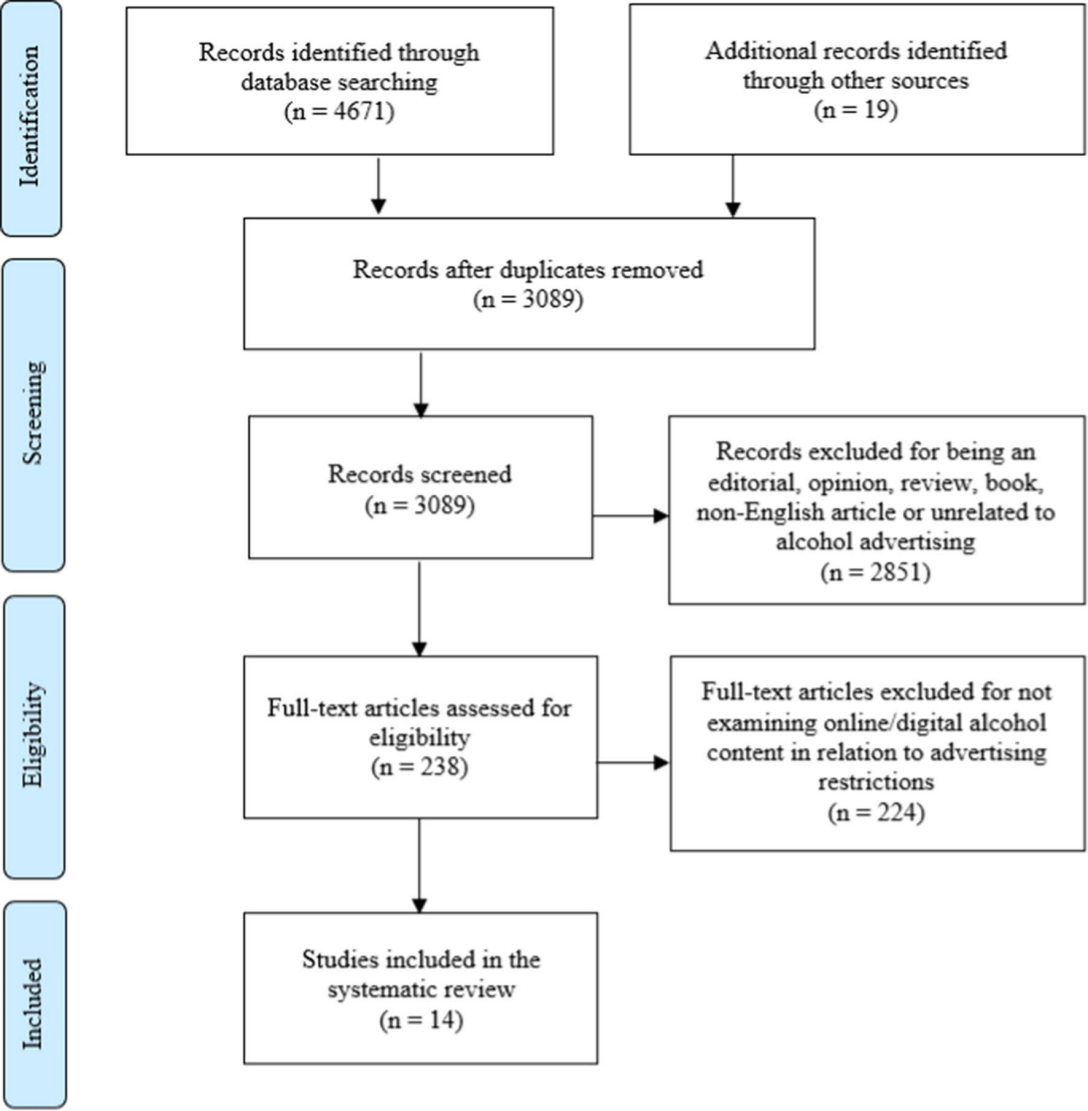
PRISMA (preferred reporting items for systematic reviews and meta-analyses) flow diagram

A combined total of 4671 records were identified through scientific database searches according to our search equation. An additional 19 articles were identified through hand-searching or reference lists’ examination. After duplicate records were removed, 3089 articles remained for further screening, out of which 2851 failed to meet the inclusion criteria, leaving 238 articles to have their texts assessed fully. The final stage, assessing eligibility led us to remove an additional 224 full-text articles. Thus, a total of 14 articles were finally included in this review (Fig. 1).

### Study Characteristics

Our systematic review identified, as mentioned, a total of 14 articles in which evidence of one or more digital alcohol advertising statutory regulations or industry self-regulatory advertising code(s) of practice violations were assessed. The selected articles were published in several types of journals in the fields of alcohol, public affairs, public health promotion, public health, and drug policy. The characteristics of the included studies are presented in Table 1, and were extracted to be relevant to our research topic of digital alcohol advertising, as multiple studies featured a mixed method design and/or broader research aims and objectives (also conducting expert or children/adolescent focus groups, assessing non-digital alcohol advertisements, etc.). Further introductory characteristics along with a shortened narrative of each study are also described the aforementioned table. Twelve studies were peer-reviewed journal papers with primary data [6, 24, 26, 30, 33–40] and two studies were gray literature reports, gathered from authoritative sources [41, 42].

**Table 1:** Characteristics and content summary of included studies

Author(s), location and study design	Types of digital media and regulation analyzed	Summary of study and findings
Mart et al. [6]	Social networking site (Facebook) Industry self-regulation	Mart et al. (2009) explored the prevalence and age-restriction systems of alcohol-related content found on the social networking site Facebook. Of the official Facebook pages of the top twelve most popular alcohol brands analyzed, six were able to be accessed by the under-aged user profile, as well as subscribe to said pages to receive direct marketing messages and promotions. Out of six alcohol applications (e.g., drinking games), four were found accessible to the under-aged profile
USA		
Cross-sectional		
Atkinson et al. [42]	Social networking sites (YouTube, MySpace, Bebo, Facebook), Website pages Industry self-regulation	Atkinson et al. (2011) as part of their mixed method study assessed the alcohol marketing content on four most predominantly used social networking sites and video file-sharing sites by UK's 8–17-year-olds (YouTube, Facebook, Myspace, Bebo) in addition to official websites of leading alcohol companies. By registering a fictitious 14-year-old user profile, no official alcohol brand pages on Facebook were able to be accessed, however only 40 (0,5%) out of 8,476 were clearly identified as official, suggesting that sites which were instead made by third parties and served as unofficial advertising of official alcohol brands, could still be accessed
UK		
Mixed method		
Gordon [33]	Website pages of alcohol companies/brands Industry self-regulation	Gordon (2011) investigated the alcohol marketing content on official websites of the top ten leading alcohol brands by sales in the UK. Of the 40 brands examined, 27 had a dedicated and/or shared website. All 27 had some sort of age-restriction entry messages or controls in place to prevent immediate access to the site. Multiple instances were also noted where the content on the alcohol brands’ websites had likely been in violation of the then-enacted regulations governing alcohol advertising in the UK, however the aforementioned regulations did not yet encompass new digital media, such as websites and social media platforms
UK		
Cross-sectional		
Brodmerkel and Carah [34]	Social networking site (Facebook) Industry self-regulation	Brodmerkel and Carah (2013) investigated fourteen major alcohol brands’ official Facebook’s pages for potential violations of the Australian alcohol industry’s self-regulatory codes of practice for alcohol advertising. According to the researchers’ findings, several alcohol brands appeared to have been in breach of a variety of self-regulatory alcohol advertising codes, such as utilizing their Facebook pages for promoting the excessive consumption of alcohol, attributing drinking to social and sexual prowess and depicting individuals under the age of 25 in their advertisements
Australia		
Cross-sectional		
Jones et al. [24]	Website pages of alcohol companies/brands Industry self-regulation	Jones et al. (2014) conducted two combined alcohol marketing-related studies, the first having assessed the effectiveness of alcohol brand websites’ own filters/entry controls, and the second having analyzed the effectiveness of commercial internet filters. Results indicated clear failures of the age affirmation systems in preventing under-aged access to digital alcohol marketing on alcohol brands’ websites. Effectiveness of commercial internet filters varied significantly as several filters appeared ineffective in blocking access, indicating the need for further software development of such internet browser extensions
Australia		
Cross-sectional		
Winpenny et al. [36]	Social networking sites (YouTube, Facebook) Industry self-regulation	Winpenny et al. (2014) analyzed the reach of marketer-generated brand content and online brand activity of five alcohol brands as well as age-gate content restriction systems on three most used social media platforms among young people in the UK (Facebook, YouTube, and Twitter). With regards to age restrictions, the under-aged profile was unable to access any of the five marketer-generated brand pages on Facebook, however, was able to successfully access as well as subscribe to all five alcohol brand channels on YouTube. The under-aged Facebook profile did not see any adverts from official alcohol brand pages after having customized his profile with interests, such as alcohol, drinking, pubs, beer, and spirits. However, it was still able to access over 70% of unofficial pages relating to drink brands, 100% of groups and events, and 96% of applications related to drink brands. YouTube and Twitter were found to not impose age restrictions on alcohol-branded content, as all examined brand channels were accessible to under-age users on both platforms. All five alcohol brands had an age verification page on their respective dedicated brand website with four out of five providing a link to the Drinkaware website in case a person inputs an age younger than 18 years old
UK		
Cross-sectional		
Barry et al. [35]	Social networking site (YouTube) Industry self-regulation	Barry et al. (2014) conducted a study, investigating the ability of under-aged access to various official alcohol brand’s channels on YouTube. Each of the under-aged profiles were able to subscribe to all 16 (100%) official YouTube channels. with two-thirds of the brands’ channels having been viewed successfully (66.67%), although the access to all channels having varied from 63 to 69% among the three user profiles
USA		
Cross-sectional		
Atkinson et al. [41]	Social networking sites (Facebook, Twitter) Industry self-regulation	Atkinson et al. (2014) conducted a three-stage study, part of which was analysis of age-restriction measures for the two social networking sites. A fictitious 15-year-old user profile was made in an attempt to access restricted alcohol brand’s official advertising content for both Facebook and Twitter. Both sites used age verification measures which prevented under-aged access, however Twitter used a self-verification barrier, which was judged to be easily by-passed by making repeated input attempts
UK		
Mixed method		
Barry et al. [37]	Social networking sites (Instagram, Twitter) Industry self-regulation	Barry et al. (2015) investigated whether alcohol companies restricted access, interaction, and exposure to marketing content on Twitter and Instagram from under-aged adolescents. All ten fictitious user profiles, both under-aged and of legal drinking age, were able to view, fully interact, and comment on advertising content from all the alcohol companies on both Instagram and Twitter, including forwarding advertisements, liking, and retweeting posts from alcohol industry feeds/pages. Out of the two platforms, Twitter’s age-gate only prevented under-aged profiles from following official alcohol brand pages, while Instagram fully allowed such advertisements to be disseminated among all under-aged profiles’ smartphones without any restrictions
USA		
Cross-sectional		
Noel and Babor [38]	Social networking site (Facebook) Industry self-regulation	Noel and Babor (2017) examined the prevalence of alcohol advertisement content and their compliance with self-regulatory alcohol advertising codes on the social networking site Facebook. Due to the overwhelming volume of alcohol advertising on said platform, the researchers focused their efforts on examining ads published by alcohol brand sponsors of the Super Bowl from one month prior and one month after the event in 2015. Out of the 50 randomly ads which were posted during the study period, 82% violated the at least one IARD guideline. 50% violated guidelines prohibiting the association of alcohol with success and health benefits. In addition, 21 thematic content areas were identified, such adventure/sensation seeking (52%), sports referencing (50%) and depicting alcohol consumption or a party atmosphere (44%). No ads contained any public health messages, although 20% contained an industry-responsibility message
USA		
Cross-sectional		
Noel and Babor [38]	Social networking site (Facebook) Industry self-regulation	Noel and Babor (2017) examined the prevalence of alcohol advertisement content and their compliance with self-regulatory alcohol advertising codes on the social networking site Facebook. Due to the overwhelming volume of alcohol advertising on said platform, the researchers focused their efforts on examining ads published by alcohol brand sponsors of the Super Bowl from one month prior and one month after the event in 2015. Out of the 50 randomly ads which were posted during the study period, 82% violated the at least one IARD guideline. 50% violated guidelines prohibiting the association of alcohol with success and health benefits. In addition, 21 thematic content areas were identified, such adventure/sensation seeking (52%), sports referencing (50%) and depicting alcohol consumption or a party atmosphere (44%). No ads contained any public health messages, although 20% contained an industry-responsibility message
USA		
Cross-sectional		
Kauppila et al. [26]	Social networking sites (YouTube, Facebook, Twitter, Instagram) Statutory regulation Industry self-regulation	Kauppila et al. (2019) conducted a study to assess the effectiveness of the Finnish 2015 law restricting alcohol marketing on social networking sites. A comparison was made between Finland and Sweden where no corresponding regulation was yet introduced at the time. Results showed that 22,5% of the Finnish and 20,2% of the Swedish social media posts by alcohol brands contained elements that could be interpreted as contravening Finnish restrictions. The content and characteristics of the posts were found to had been overwhelmingly within the scope of self-regulatory guidelines, with only seven (0.006%) of the 1204 posts having breached the self-industry alcohol marketing codes. Out of alcohol brands with registered Facebook accounts, only 62% Swedish and 63% Finnish sites had activated age limits to prevent under-aged access only 13% of Instagram accounts were restricted in Finland, meanwhile no age-checks at all were found on the Swedish brands' Instagram accounts
Finland		
Comparative audit		
Paradis et al. [40]	Social networking site (Facebook) Statutory regulation Industry self-regulation	Paradis et al. (2020) evaluated the scope of potential violations of Canada’s statutory alcohol advertising code (CRTC Code) by 58 different drinking venues, as well as assessing, whether said violations relate to their popularity among different university’s students and their drinking behaviors. A sample of 477 university students were tasked with assessing alcohol-related social media posts by drinking venues, ranked by the research team to be as most conflicting with the 17 CRTC Code guidelines. Evaluations indicated that for 15 out of the 17 guidelines, as little as 1,7% and no more than 46,6% of drinking venues posted alcohol-related content which adhered to the CRTC Code. Compliance by the drinking venues was also investigated for associations to students’ drinking behavior, and evidence suggested a clear association between students, who were classified as heavier drinkers, preferring alcohol-related content by less compliant drinking venues
Canada		
Cross-sectional		
Barry et al. [39]	Website pages of alcohol companies/brands Industry self-regulation	Barry et al. (2020) examined the effectiveness and the type of age gates preventing access of underage users to official websites of the top 25 alcohol brands in the USA. Out of the 23 websites 100% contained an age gate / age verification page. By providing an underage birth date, 100% of the pages restricted access to the website, however only 22% of them blocked future attempts, and out of the remaining 78%, 56% still allowed entry if a newly 21 + years of age date of birth followed the initial under-aged inputs
USA		
Cross-sectional		
Pierce et al. [30]	Social networking sites (Facebook, Instagram) Industry self-regulation	In their study, Pierce et al. (2021) assessed the age-restriction controls on Australia's most dominant alcohol companies' official brand Facebook and Instagram account profiles. Despite the Australian alcohol companies having to abide by the self-regulatory Alcohol Beverages Advertising Code (ABAC) Scheme, which requires the use of age-restriction controls on alcohol brand accounts on social networking sites, 28% of Instagram and 5% of Facebook accounts in the study were found to be accessible to young people under the legal age of 18 years
USA		
Cross-sectional		

### Industry Self-Regulation – Effectiveness of Age Affirmation Systems

Eleven studies have, as the main or partial research focus, assessed the effectiveness of age affirmation (age-gate) mechanisms, which are imbedded into brand websites or social networking sites and image/video sharing platforms as an industry self-regulatory measure with the purpose of preventing under-aged persons from being directly exposed to alcohol advertising content on digital media. Out of the eleven studies, three have assessed solely age affirmation mechanisms of alcohol brand owned websites [24, 33, 39], seven solely social media platforms and/or image/video sharing platforms [6, 26, 30, 35–37, 41], and one study assessed the aforementioned age gates on both types of digital media [42]. All studies which assessed systems on social media and related platforms, were similar in their methods and design by having created one or more fictitious user profiles and assigned them with various ages, at least one being of under the legal drinking age in a respective country. Trials were then conducted on the sites, whether the fictitious under-aged user accounts were able to access, interact, share, and/or subscribe to alcohol advertising content. Most studies examined Facebook or Twitter. For the three studies which assessed age gates on alcohol brand’s websites, similar trials of attempting to breach the site were used, by either inputting different ages, and then analyzing if the mechanisms prevented access to under-aged users, blocked repeated attempts, displayed alcohol health warning messages, and/or deferred under-aged users to a 3rd-party website with alcohol-related harm prevention services. The results point toward existing age-gate measures being largely ineffective in preventing access to children and under-aged adolescents with inconsistencies found on every single examined platform and website. Only in a single study did age gates prove to be mostly successful [41], whereby in others e.g., [6, 24, 37, 39], alcohol advertisements were accessible to most if not all users under-aged users. Alcohol brands’ websites featured even less-effective age gates in comparison to social media. Commercial internet filters also allowed access to one-third of ‘prohibited’ websites [24].

### Industry Self-Regulation – Compliance of Alcohol Advertising Content

Five studies have as a main focus or as a secondary research objective analyzed digital alcohol advertising content posted by user profiles of alcohol brands or their related 3rd parties on social media and/or image/video sharing platforms and assessed them for compliance with the alcohol industry’s self-regulatory codes of practice. Studies observed and analyzed advertising content on Facebook pages [34, 38, 42], Twitter [41], and alcohol brands’ own websites [33]. Cases of violations of self-regulatory advertising codes, which were identified by the authors, related to alcohol brands posting content which promoted the excessive consumption and attributed alcohol use to social and sexual prowess [34], another found alcohol brands posting advertisements real-life events, quizzes and competitions, price promotions, and encouraged users to share, like, retweet alcohol brands’ status/posts [41]. Advertising themes whereby the alcohol profiles took advantage of certain aspects of youth culture and appeal (e.g., associating drinking to dangerous and irresponsible behaviors) in promoting alcoholic beverages, were also noted [33]. Lastly, Noel & Babor’s [38] findings showed poor self-regulatory adherence of alcohol brands’ Facebook ads in their limited data sample with 82% of alcohol brand advertisements violating at least one guideline, e.g., promoting irresponsible consumption, social success as a consequence of alcohol use, connecting alcohol to potential health benefits, and even including some explicit (e.g., sexual) code violations. This although limited assortment of empirical evidence, although being unable to be generalized, supports the notion that self-regulatory alcohol advertising codes on digital media may often be of limited effectiveness and require further attention from policymakers.

### Statutory Regulation – Effectiveness and Impact of Legislative Change

Only two studies we retrieved [26, 40] analyzed the effectiveness of statutory regulations governing digital alcohol advertising. Both assessed compliance of posted alcohol advertising content on social networking sites. However, one analyzed content posted by drinking venues near Canada’s major universities on Facebook and Instagram against potential violations of the Canadian Radio-television and Telecommunications Commission Code (CRTC) Code [40], and the second study primarily analyzed advertising content posted by major Finnish alcohol producers’ profiles on Facebook, Instagram, Twitter and YouTube against potential infringements of the Finnish Alcohol Act, which was amended in 2015 to also cover restrictions of user-generated content in social media marketing. In the Canadian study, it was found that out of a sample of 58 drinking venues, no more than 27 (46,6%) of venues posted alcohol advertising content on their social media pages which adhered to the CRTC Code. With regard to the Finnish study [26], proper adherence of sampled social media posts in 2014 (before the law was enacted) was found to be 76%, which increased to 84% in the 2017 sample. Likely youth-appealing elements (e.g., sports activities, use of celebrities, humor, relaxation, etc.) were still prevalent in some social media posts. However, the authors deemed almost all as being published without the intention of specifically targeting young or even under-aged audiences.

### The Quality of Studies

With regard to the assessment criteria, the quality of included studies varied slightly (Appendix 1) with most studies being deemed to be of high quality. Out of a maximum score of 36 on the Hawker et al. [32] quality appraisal scale, all studies except one had reached a score of 30 or higher. One study reached a full score of 36/36, and the lowest scoring study reached a 28/36. Across the studies, the highest scored criteria were for the Introduction and Aims (98%), Sampling (98%), Findings/Results (98%) as virtually all offered easy to understand findings, written in logical progression and their results related directly to the study aims, Transferability or Generalizability (96%) as studies generally described their context and settings sufficiently to allow comparisons with others, and Implications and Usefulness (98%) as all but one suggested ideas for further research, and suggested implications for alcohol advertising policies, etc. The lowest sub-scores were noted the Ethics and Bias (46%) as multiple studies were deemed not to have properly addressed or were unreflective of the likelihood of researcher bias, and the Data Analysis (78%) as multiple studies did not perform their data analysis to the point, which would be regarded as sufficiently rigorous and/or did not discuss statistical implications of their results. Overall, a high consensus among reviewers was noted as only a few minor disagreements arose with regards to a few of the studies.

## Discussion

To our knowledge, this review is the first in the field of digital alcohol advertising to systematically focus on the impacts and effectiveness of statutory regulation, co-regulation, and/or self-regulation frameworks of alcohol advertising and marketing practices on social networking/image sharing sites, online video hosting/sharing platforms and official alcohol industry/brand websites. An immediate finding and a simultaneously worrying result of our review was the fact we were only able to retrieve a single study, conducted within the EU region, which had assessed the impact of legislative change following the enactment of a country’s new statutory restrictions, which regulate digital alcohol advertising content on social networking sites [26]. The other article, describing potential violations of statutory alcohol advertising restrictions (Canadian CRTC Code) was by Paradis et al. [40], however their research scope focused solely on a narrow assortment of social media posts by drinking venues in one of the Canadian provinces. This sort of a lack of wide-scale empirical evidence makes it all but impossible to reach unequivocal international implications and comparisons. It is also difficult to reach any generalizable conclusions due to the fact that almost all of the online video sharing/hosting platforms and social networking/image sharing sites, which were assessed in our retained studies, have amended their advertising policies, have changed/updated their sign-up methods and restrictions, as well as age affirmation mechanisms multiple times over the last decade.

A substantial amount of the selected studies was also the first in their fields to undertake such empirical research. To our knowledge, Mart’s study [6] was the first peer-reviewed study to assess the prevalence of alcohol-related content and the effectiveness age restrictions found on Facebook, and according to their respective authors, Barry et al. [35] study was the first study attempting to describe the accessibility of alcohol content on YouTube to underage persons in the USA, Winpenny [36] was the first study attempting to describe the exposure of children and young adults to alcohol marketer-generated content on social media websites in the UK, Noel and Babor [38] conducted the first study to systematically evaluate alcohol advertising on social media for compliance with the content guidelines of a self-regulated alcohol advertising code, and Pierce et al. [30] was the first study that has examined the use of age-restriction controls by alcohol companies on Facebook and Instagram in Australia.

Regarding the effectiveness of age affirmation technology on social networking sites or on alcohol brands’ official websites, a consistent trail of exploratory evidence from the oldest included study by Mart et al. 2009 [6], all the way to the newest by Pierce et al. 2021 [30] suggest that the alcohol industry and social networking sites, despite the available technology, have been largely inconsistent at implementing effective age-restriction measures on their platforms, which would prevent under-aged adolescents from accessing alcohol advertising content. In some cases [41] age-restriction measures for Facebook were successful, yet in other cases, as noted by Jones et al. [24], mechanisms were so poorly implemented, that access was being granted to an alcohol brand’s website, regardless of any date of birth provided. In the case of Barry et al. 2014 [35], under-aged profiles were able to subscribe to all of the 16 identified alcohol brands’ YouTube channels and two-thirds of the brands’ channels were successfully viewed, and in Barry et al. 2020 [39], all under-aged user profiles, were able to view, fully interact, and comment on advertising content from all the alcohol companies on both Instagram and Twitter. Winpenny’s et al. [36] findings also highlight that incorrect ages are often given on the Facebook profiles by younger children to avoid having denied access to a social networking site. Therefore, it can be assumed that age-affirmation mechanisms, may only be effective in blocking accidental access to websites promoting alcohol, but it cannot be reasonably expected to withstand simple and repeated efforts of an adolescent with enough time and average computer skills, a conclusion similarly echoed by Barry’s et al. 2014 [39] study findings. As noted by Pasquale et al. 2022 [43] a possible solution to more robust age affirmation mechanisms on social networking sites would be the limited use of biometrics features. However, adopting such approaches should be considered very carefully, as their implementation could pose a serious risk of potential misuse of private user data collection and may go against current data protection regulations. To combat public concerns, some companies such as Instagram have allegedly begun using various proprietary techniques to identify children who have entered fake information as part of the sign-up process, the specific details of these mechanisms, however, are kept confidential to prevent them from being bypassed [44] and we were unable to find any publicly available evidence of them being assessed for effectiveness.

Our review’s findings point to the consistent, however substantially limited empirical evidence of the ineffectiveness of industry self-regulatory restrictions of alcohol advertising content. As noted by other research concerning traditional alcohol advertising [45–47], despite imposed regulations limiting exposure of youth to alcohol advertising content, evidence exists of the alcohol industry likely targeting those under the minimum legal drinking age. In the case of Brodmerkel and Carah [34], alcohol brands routinely promoted excessive consumption of alcohol, attributed social and sexual prowess to the consumption of alcohol and publish content depicting under-aged individuals, doing so in violation of their own self-regulatory codes of practice for social networking sites. In their study, Mart et al. [6] reached a conclusion that Facebook did not appear to monitor or ensure compliance with its own alcohol advertising rules. As identified by Gordon [33] in numerous instances, the thematic content on alcohol brands’ official websites likely went against the then British alcohol advertising codes of practice, as alcohol brands posted advertisements which could be considered as appealing to people under the age of 18 by taking advantage of certain aspects of youth culture and appeal, as well as associating drinking to dangerous and irresponsible behaviors. Atkinson et al. [41] also identified alcohol brands as taking advantage of social networking sites to routinely promote real-life events, quizzes and competitions, price promotions, encouraging users to share, like, retweet alcohol brands’ status/posts, and also encouraging alcohol use. These findings are in line with more recent evidence [48] which affirms alcohol companies still use thematic content on their digital advertisements, which are known to be attractive to children and young people. In a recent study, which was published too late to be retrieved for our review, by Jongenelis et al. [49], 94% of the 628 analyzed Australian alcohol ads were found violating at least one AARB Code provision, indicating ineffectiveness of the ABAC self-regulatory codes. Another study by Russell et al. [50], analyzing top 100 popular videos on TikTok with the hashtag #alcohol, found 98% expressed pro-alcohol sentiment and 69% conveyed positive experiences of alcohol, thus demonstrating a propensity to promote rapid consumption of alcohol drinks, likely going against the platform’s alcohol advertising policies. After our systematic review was already finished, a relevant Lithuanian case study was published in the second half of 2022 [51], which assessed the effectiveness of their comprehensive statutory alcohol advertising ban (including digital alcohol advertising restrictions). Content on 110 popular Facebook and Instagram pages was assessed to determine compliance with current legislation, and found extremely high compliance across both platforms, as only 1,4% of 2000 + analyzed social media posts were deemed to be alcohol advertisements (infringing on the Alcohol Control Law).

### Implications for Policy and Research

With regard to answering our research questions, currently available peer-reviewed as well as gray literature evidence, both of which are substantially limited, it points toward the direction that both past and current regulatory policies have proven themselves to be often ineffective in protecting adolescents, young adults, adults, and vulnerable groups from digital alcohol advertising practices. A multitude of challenges regarding digital alcohol advertising restrictions remain to be solved by policy-makers. As per the report of the WHO Regional Office for Europe, the enactment of digital advertising regulation should ideally be supported and accompanied by effective systems of enforcement and monitoring, which would transparently investigate compliance and adjudicate complaints. As noted by Critchlow et al. [52], updated marketing codes have to account for user-generated branding, as well as other contemporary and emerging marketing techniques, such as digital sponsorships, augmented reality, blogs and vlogs, sweepstakes and drinking games, etc. Regulators also need to address, how brands use cultural life and people’s identities for social media activity and promotions [34], as they use strategies to stimulate the integration of the brand and alcohol consumption in the mediation of everyday life and amplify people’s cultural identities, as well as prompting consumers to say things that brands are prohibited from saying, therefore circumventing the spirit of the self-regulatory codes of conduct. Additionally, a unified transnational approach and inter-governmental cooperation would likely result in an easier and more comprehensive legislative transition. Ideally, future regulations should also be drafted in a manner which best encompasses and pre-empts the rapid advancements of digital media (e.g., artificial intelligence, virtual reality (e.g., ‘metaverse’). Although not a direct finding of our results, we argue that continuous and increased regulatory scrutiny should not solely be placed on the alcohol industry, but also the social network service providers themselves with regards to their search and advertising algorithms, as well as how consumer data is gathered, processed, and utilized, as there is a complete lack of transparency and an overall reluctance to share such information with the general public nor legislative policy-makers.

It is of crucial importance that scientific research assessing the effectiveness of digital alcohol marketing restrictions, regardless of the regulatory framework approach, continues to be supported, because as evident, major gaps in knowledge remain in this research field with regards to not only assessing digital advertising legislation of EU member states, as well as other world countries. Research needs to also expand and employ longitudinal and experimental designs to clearly make evidence-based assertions about causal impacts of legislative changes. Other mixed methods such as conducting focus groups or interviews, would also be recommended as a complimentary study design. However, such research would be considerably more resource-demanding. Considering the rise of social media influencers and known inconsistencies regarding alcohol sponsorships transparency in recent years as well as their ability to promote or undermine products [53] as well as keeping in mind many minors can be exposed to such ‘alcoholposts’, potentially leading to increased drinking among vulnerable age groups [23], future research should also explore how collaborations with influencers are utilized by the alcohol industry.

### Strengths and Limitations

There are several strengths and limitations of this review, which should be considered. First, the review expands on the scope of the Lobstein et al. [24] limited narrative review by conducting a more detailed analysis, including a quality assessment of its included studies on top of which we present the most recent evidence on this topic up until 2021. Despite a relatively modest sample of 14 studies, we present a geographically diverse overview of literature from seven different countries. Regardless of the substantial heterogeneity of the included studies, we applied a detailed methodological approach and rigor to ascertain the quality of the retrieved scientific as well as authoritative gray literature sources. Both review phases of the initial title/abstract eligibility assessment as well as the full-text assessment were conducted independently by two researchers with a third researcher making the final decision in an event of any inclusion/exclusion disagreements. The same process was undertaken for the study quality appraisal portion of the review. We also submitted a methodological protocol for registration on the PROSPERO database, which was subject to external expert review, prior to its submission. A number of limitations can be identified. We conducted our main search from the year 2004 to mid-2021, therefore it is possible, that some studies which are relevant to our research topic, published prior or after the cut-off date and which were not identified via subsequent manual searches and therefore could have been missed. The review included articles published in English only, and relevant non-English publications may have been overlooked since their respective authors, whom we contacted, either were unable to provide us with a basic description in English of the study’s objectives and findings or were unresponsive in our communication attempts. Although our research includes an overview from several countries, all but one study included in our review originated from English-spoken countries and only one from non-English-spoken country. Our search was limited by chosen key words and journal databases. From 4671 records identified through scientific database searches and additional 19 articles identified through hand-searching or reference lists’ examination, relatively modest samples of 14 articles were finally included in this review. Due to almost all studies having reached a very high-quality assessment score (and this being a common occurrence with the tool in other published studies also), using a different tool with an even more rigorous appraisal process is suggested. A broader limitation of the evidence base can be noted regarding the absence of identified longitudinal studies, as our studies largely chose a cross-sectional approach, which cannot be used to infer clear causality. Additionally, we found a lack of statistical rigor/conclusions as part of the data analyses in some of the included studies.

## Conclusions

Current empirical research, although still limited, suggests that digital alcohol advertising content remains widespread on social networking and image sharing sites, online video hosting/sharing platforms, and regular websites, including those currently most popular by vulnerable groups and young adults. As evident by our systematic review, young people including under-aged adolescents, who are the most prominent users of such platforms, continue to be targeted by as well as being able to interact with alcohol advertising content, indicating a clear failure of existing industry self-regulatory policies along with their age-affirmation mechanisms, which often tend to be largely ineffective at preventing prolonged and determined under-aged access attempts, despite significant technological progress having been made in the last decade. The alcohol industry continues to use increasingly innovative marketing strategies, not yet covered by statutory regulation frameworks, to reach their targeted audiences. Digital marketing and advertising of alcohol content, which is being spread through social media and image/video sharing platforms by alcohol producers and their brands, is often inconsistent with their own industry self-regulation frameworks. There is a clear need for current voluntary alcohol advertising codes of practice to be updated, monitored and enforced in a transparent manner by either governmental bodies or organizations, independent from the alcohol and advertising industries. A substantial amount of research throughout all world regions, especially in a longitudinal design setting, is needed to establish strong causational evidence and generalizable correlations with regards to assessing the effectiveness and impacts of new/updated regulatory frameworks on contemporary digital alcohol advertising practices.

## Supplementary Information

Below is the link to the electronic supplementary material.

Supplementary file1 (DOCX 22 KB)

## Data Availability

Not applicable.
