# Supplementary material for: Effectiveness of Regulatory Policies on Online/Digital/Internet-Mediated Alcohol Marketing: a Systematic Review
Source: J Epidemiol Glob Health. 2023 Feb 2;13(1):115–28. doi: 10.1007/s44197-023-00088-2 (PMC10006384; doi:10.1007/s44197-023-00088-2)
Supplement: Supplementary file 1 — Supplementary file1 (DOCX 22 KB) [file 44197_2023_88_MOESM1_ESM.docx]

**Appendix 1:** Quality Assessment of Included Studies

| **Studies** | **1** | **2** | **3** | **4** | **5** | **6** | **7** | **8** | **9** | **Score (out of 36)** |
| --- | --- | --- | --- | --- | --- | --- | --- | --- | --- | --- |
| Mart et al. (2009) | 3 | 3 | 4 | 4*^1^ | 3 | 1*^2^ | 4 | 4 | 4 | **30** |
| Atkinson et al. (2011) | 4 | 4 | 4 | 4*^1^ | 3 | 3*^2^ | 4 | 4 | 4 | **35** |
| Gordon (2011) | 4 | 4 | 4 | 4*^1^ | 3 | 1*^2^ | 4 | 4 | 4 | **34** |
| Brodmerkel & Carah (2013) | 3 | 4 | 3 | 3*^1^ | 3 | 1*^2^ | 4 | 3 | 4 | **28** |
| Jones et al. (2014) | 3 | 4 | 4 | 4*^1^ | 3 | 1*^2^ | 4 | 4 | 4 | **31** |
| Winpenny et al. (2014) | 4 | 4 | 4 | 4*^1^ | 3 | 3*^2^ | 4 | 4 | 4 | **34** |
| Barry et al. (2014) | 4 | 4 | 4 | 4*^1^ | 3 | 1*^2^ | 4 | 4 | 3 | **31** |
| Atkinson et al. (2014) | 4 | 4 | 3 | 4*^1^ | 3 | 1*^2^ | 3 | 4 | 4 | **30** |
| Barry et al. (2015) | 4 | 4 | 4 | 4*^1^ | 3 | 1*^2^ | 4 | 4 | 4 | **32** |
| Noel & Babor (2017) | 4 | 4 | 4 | 4*^1^ | 4 | 4*^2^ | 4 | 3 | 4 | **35** |
| Kauppila et al. (2019) | 4 | 4 | 4 | 4*^1^ | 3 | 3*^2^ | 4 | 4 | 4 | **35** |
| Paradis et al. (2020) | 4 | 4 | 4 | 4 | 4 | 4 | 4 | 4 | 4 | **36** |
| Barry et al. (2020) | 4 | 4 | 4 | 4*^1^ | 3 | 1*^2^ | 4 | 4 | 4 | **32** |
| Pierce et al. (2021) | 4 | 4 | 4 | 4*^1^ | 3 | 1*^2^ | 4 | 4 | 4 | **32** |

| **Criteria:** | 1. Abstract and title  2. Introduction and aims  3. Method and data  4. Sampling  5. Data analysis  6. Ethics and bias  7. Findings/results  8. Transferability/generalizability  9. Implications and usefulness | **Rating Index:** | 4 points = Good  3 points = Fair  2 points = Poor  1 point = Very Poor | **Notes:** The quality appraisal was conducted independently by two reviewers and the total score represents the average of the two scores, where applicable. *^1^ denotes a study which analysed various datasets in lieu of directly assessing human participants, necessitating an adaptive scoring. In such cases a *^2^ denotes ethics research approval considerations as not having been assessed. |
| --- | --- | --- | --- | --- |
